# The challenges in diagnosing isolated epididymal tuberculosis (TB) in an adolescent male: a case report

**DOI:** 10.1186/s12894-024-01442-7

**Published:** 2024-03-19

**Authors:** Citra Cesilia, Harry Galuh Nugraha, Safendra Siregar, Heda Melinda Nataprawira

**Affiliations:** 1 Division of Respirology, Department of Child Health, Faculty of Medicine, Universitas Padjadjaran/ Hasan Sadikin General Hospital, Pasteur Street No. 38, Sukajadi, Bandung, West Java 40161 Indonesia; 2 Department of Radiology, Faculty of Medicine, Universitas Padjadjaran, Hasan Sadikin General Hospital, Pasteur Street No. 38, Sukajadi, Bandung, West Java 40161 Indonesia; 3 Department of Urology Surgery, Faculty of Medicine, Universitas Padjadjaran, Hasan Sadikin General Hospital, Pasteur Street No. 38, Sukajadi, Bandung, West Java 40161 Indonesia

**Keywords:** Adolescent, Epididymal TB, Extrapulmonary TB, Genitourinary tuberculosis, Teratozoosperma

## Abstract

**Background:**

Genitourinary tuberculosis (GUTB) is a common form of extrapulmonary TB (EPTB) in children. An example of GUTB is epididymal TB, which usually presents unspecific chronic clinical manifestations. Definitive diagnosis can be conducted based on bacteriologic confirmation and histopathologic results, but this is challenging due to the paucibacillary nature of EPTB. Therefore, we reported the challenges in diagnosing isolated epididymal TB in an adolescent male.

**Case presentation:**

A 16-year-old male presented to respirology clinic with painful swelling of the left scrotum for 3 months before visiting to the hospital. The symptoms were associated with persistent coughing for 2 months, and physical examination of the left scrotum showed swelling accompanied by cardinal signs. A palpable hard mass was found on the left scrotum, with firm borders, measuring 7 × 4 cm. Laboratory examination and tumor markers were within normal limits, although leukocyturia was found, and the urine culture was negative. Genital ultrasound (US) showed epididymitis sinistra with septal hydrocele, while magnetic resonance imaging (MRI) indicated inhomogeneous left epididymitis with bilateral inguinal lymph node enlargement. Although TB evaluation presented a negative purified protein derivative (PPD) test and bacteriologic examination, chest X-ray (CXR) showed perihilar lymphadenopathy. Based on the clinical and radiologic results suggesting TB, the patient was diagnosed with isolated epididymal TB and received quadruple antituberculosis therapy (ATT) for 6 months. After treatment, the left testicle size started to shrink and was equal to the right testicle, also, there were no signs of inflammation, the body weight increased by 5 kg, and cough disappeared. Sperm analysis at the end of treatment indicated teratozoospermia, which was subsequently treated by the urologic surgery department.

**Conclusions:**

Biopsy and bacteriologic confirmation for TB epididymitis were challenging to perform in the clinical setting. Epididymal TB should be considered in adolescent males with complaints of chronic scrotal swelling and pain. Clinical judgment based on history taking, physical examination, and radiologic features supporting TB features could be helpful in accurate and fast diagnosis for favorable outcome.

## Background

TB in children and adolescents is recognized as a worldwide health problem, [1] spreading to almost all organs, through the respiratory system as the main route [2]. Based on CXR results, Pulmonary tuberculosis (PTB) is only found in < 50% of EPTB. In adults, EPTB reportedly accounts for 5−45% of total TB cases, while GUTB is responsible for 4% [3, 4]. GUTB is defined as infectious inflammation of urogenital system organs in any combination, caused by *Mycobacterium tuberculosis* (*M. tuberculosis* ) or *Mycobacterium bovis* [6]. Several types have been reported including kidney, urinary tract, epididymal-orchitis, and prostate TB [6]. The typical pathways of infection are hematogenous seeding, intracanalicular or direct extension from nearby foci on the genital tract, and infection descending from the kidneys [4]. Due to unspecific symptoms and a lengthy latency between the initial infection and clinical manifestations, diagnosis is challenging. According to previous studies, GUTB also mimics other testicular lesions in most situations [4, 6]. 

The incidence of GUTB in children, both in Indonesia and globally, is unknown [4]. Studies on adult cohort reported increased GUTB in males from 5.5 to 25.3% between 2006−2019 in several countries [5, 6]. Case diagnosis is often very difficult except in patients with high TB suspicion [6]. One type of GUTB, namely epididymal TB, [3–8] has been reported in children with the oldest known case dating back to 1963 [9, 10]. 

This study presented a case of an adolescent male with epididymal TB based on clinical and radiological results without bacteriological confirmation. Despite the complexity of the case due to paucibacillary characteristics in pediatric TB, this study offers insight into the challenges of clinicians in establishing the diagnosis of epididymal TB in children.

## Case presentation

A 16-year-old male complained of painful swelling on the left scrotal for the last 3 months before visiting respirology clinic in April 2023. This lump had first appeared on the left scrotum approximately a year before admission (February 2022). However, the pain persisted even after receiving paracetamol 500 mg q.i.d orally from the community health center for 1 week. The complaint was accompanied by a persistent cough for 2 months and no weight gain in the last year. There were no complaints of fever, urinary problems, or history of trauma to the genital area. In February 2023, the patient was taken to the regional hospital and treated for 4 days, receiving ceftriaxone 80 mg/kg/day intravenously, and the genital US showed left epididymitis. There were no TB contacts identified, and no history of drug abuse, smoking, or active sexual intercourse. The patient was well-nourished, had not been given the BCG vaccine, and lived in a district 13.2 km from the capital city, with low-income family.

Genital examination showed an enlarged left scrotal, palpable mass with hard consistency, flat surface, firm borders, measuring 7 × 4 cm, pain, and redness. The transillumination test was negative, while multiple bilateral, enlarged lymph nodes with a 1−1.5 cm diameter were palpated in the inguinal region. Other physical examinations were within normal limits.

**Fig. 1:**
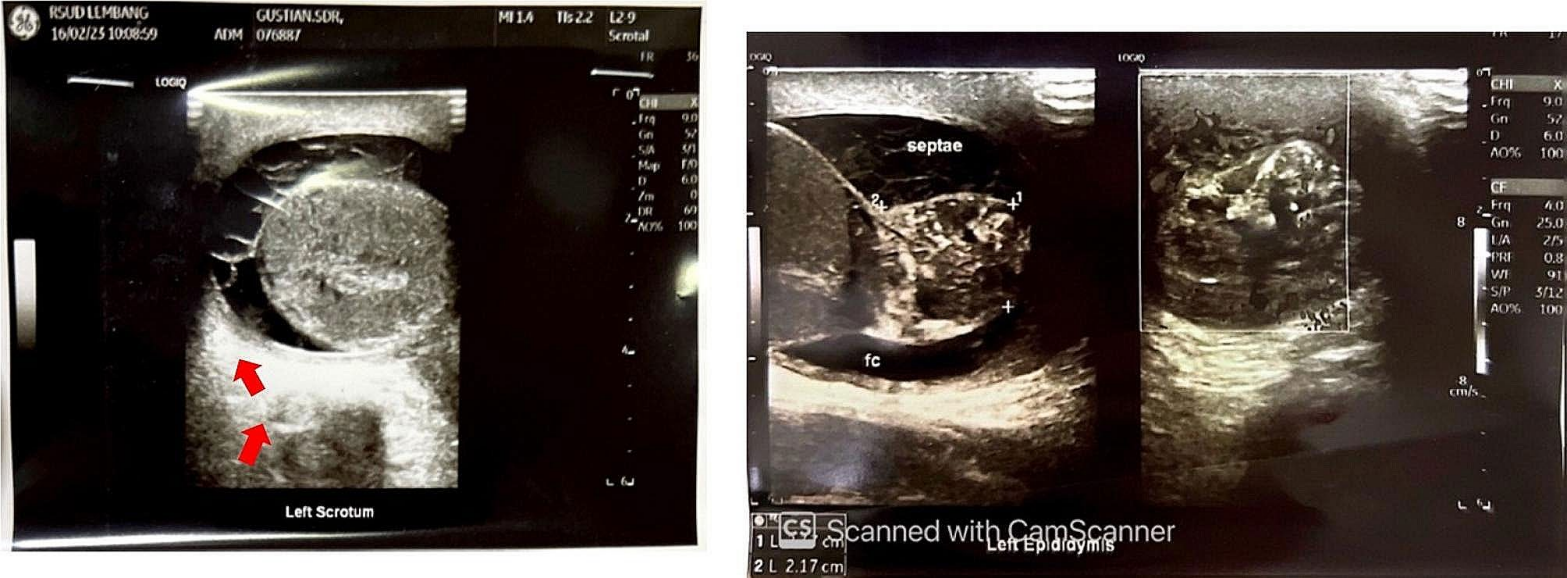
Previous testicular US suggestive of left epididymitis accompanied by septae hydrocele (red arrow)

**Fig. 2:**
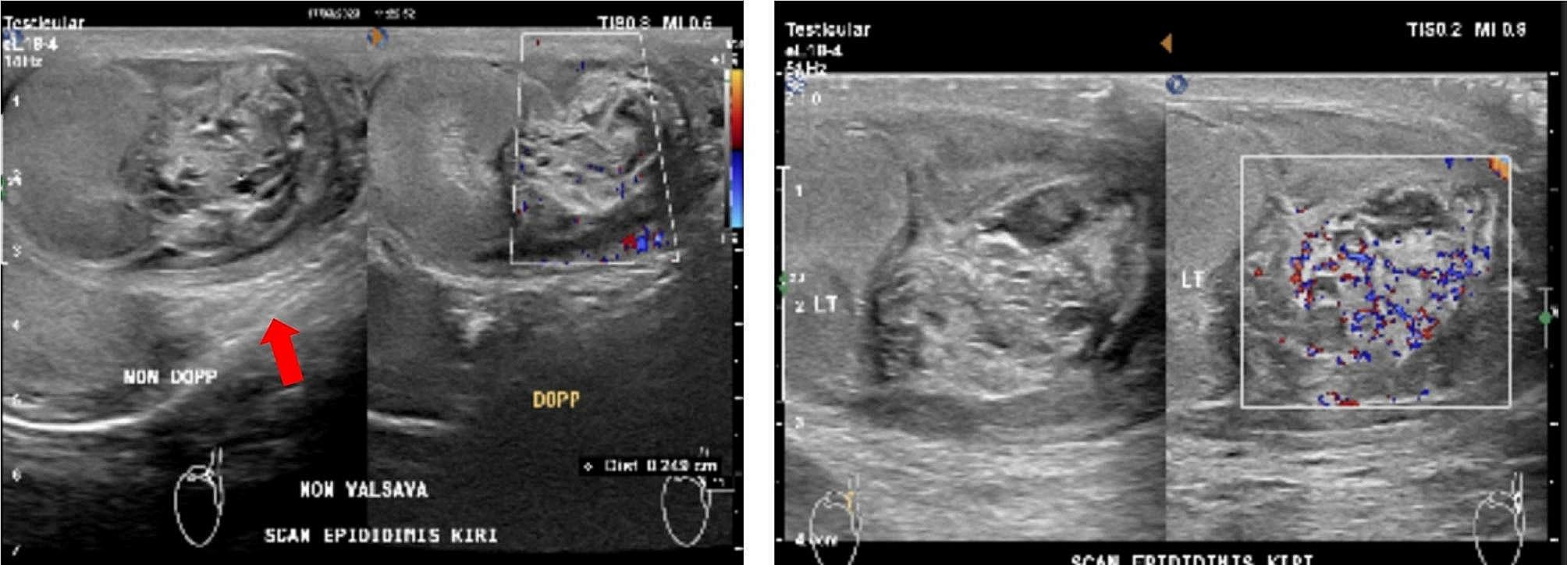
US testis suggestive of grade II – III varicocele with enlarged left epididymitis (by left testicular inhomogenicity (Red arrow)

**Fig. 3:**
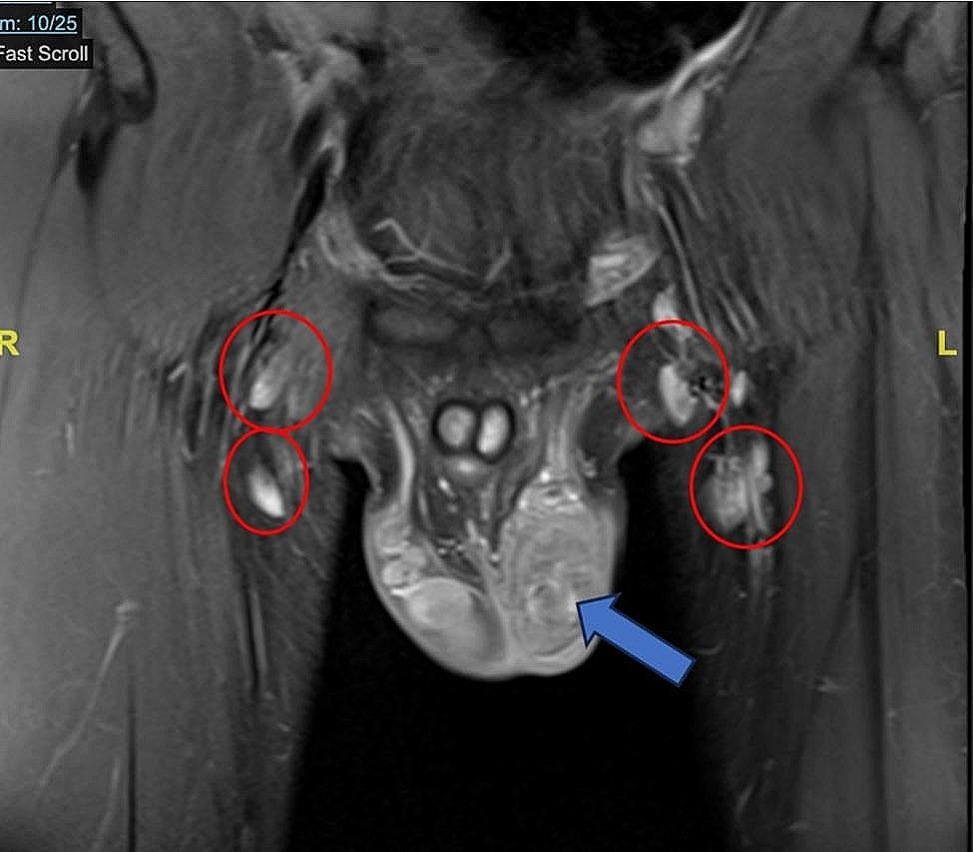
Genital MRI with contrast showed enlargement of epididymis. Post-contrast scanning provides inhomogeneous enhancement (Blue arrow) and enhancement of inguinal ring (Red circle)

Routine blood test results were within normal limits, anti-HIV was negative, and tumor markers (including alpha-fetoprotein/AFP, beta-human chorionic gonadotropin/ Beta HCG, lactate dehydrogenase/ LDH) were within normal limits. Urinalysis presented leukocyturia, while urine culture for both bacterial and *M. tuberculosis* was negative. PPD test was negative, but CXR showed perihilar lymphadenopathy due to TB. Furthermore, acid-fast bacilli (AFB) smear, GeneXpert MTB/Rif, and *M. tuberculosis* culture examination of induced sputum had negative results, well as GeneXpert MTB/Rif from urine. The available supporting examination data from the previous hospital included testicular US which was suggestive of epididymitis sinistra with septal hydrocele and suspected left scrotal hernia (Fig. 1). The testicular US was repeated at our hospital 2 months after the previous one, and the results suggested grade II−III varicocele with enlarged left epididymitis (post-infection) and inhomogeneity of the left testicle (Fig. 2). The patient was subjected to genital magnetic resonance imaging (MRI) which was suggestive of left epididymal-orchitis and bilateral multiple inguinal lymph node enlargement, with no hydrocele, varicocele, or signs of malignancy. (Fig. 3).

**Fig. 4:**
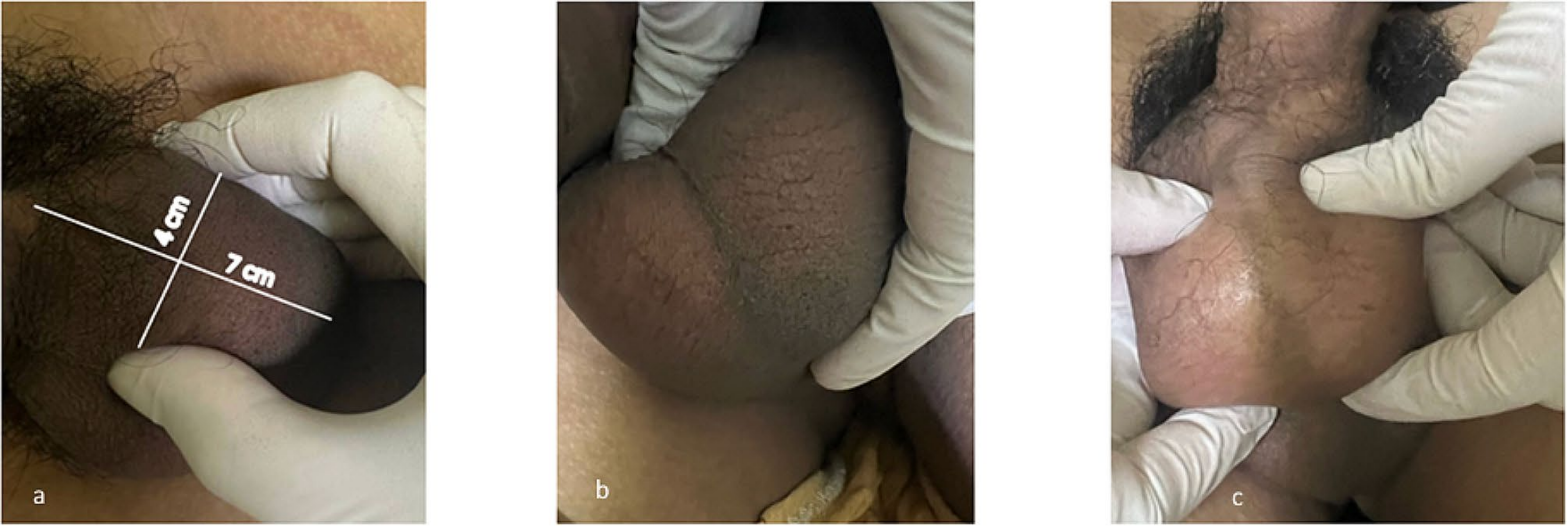
Left testicle enlargement showed at first visit (a), fourth month of treatment (b), and sixth month of treatment (c)

Based on the examination results, the patient was diagnosed with isolated epididymal TB and treated with an adult Fixed-Dose Combination (FDC) 4 tablets per day. This combination consisted of isoniazid 75 mg/-rifampicin 150 mg/-pyrazinamide 400 mg/-ethambutol 275 mg for 2 months, then continued with isoniazid 75 mg/-rifampicin 150 mg for 4 months daily. After treatment, clinical improvements were found in weight gain of 5 kg (from 52 to 57 kg), improved appetite, as well as no complaints of coughing and left scrotal swelling. There were no side effects during the treatment, and at the last physical examination, the size of the testicle had decreased from 4 × 7 cm to 3 × 2 cm, similar to the right testicle, and no signs of inflammation were found. (Fig. 4) However, sperm analysis results at the end of treatment showed the presence of teratozoospermia, which remained under therapy.

## Discussion and conclusions

The prevalence of GUTB in children remains unknown, despite the earliest report dating back to 1963 for epididymal TB [9, 10]. Examination modalities with high sensitivity and specificity to diagnose epididymal TB are still limited, along with non-specific clinical symptoms. Consequently, this disease is often misrecognized as a bacterial infection or tumor [11]. 

Epididymal TB in adults is caused by retrograde spread from the prostate and seminal vesicles or through a continuum transmission from nearby organs [4, 6, 11]. However, in children, this disease results from hematogenous spread [6, 7]. The prostate and epididymis are the most commonly affected sites, although other organs such as the testes, vas deferens, scrotum, seminal vesicles, urethra, and penis may also be affected [12]. Epididymal TB may occur without clinical or laboratory evidence of renal association, known as isolated epididymal TB [13]. This condition is rare, accounting for approximately 20% of GUTB, and challenging to diagnose [13–15]. 

The patient in this report was 16 years old at diagnosis, and a study conducted for 11 years in China showed that the average age of epididymal TB was 50.77 ± 16.1 years (with a range of 13−90 years) [11]. In this report, the diagnosis was made from clinical symptoms in the form of gradual swelling and pain in the left pubic pouch 3 months ago, which was also previously felt one year ago. Clinically, epididymal TB usually shows symptoms of gradual scrotal swelling accompanied by pain, although there may be no pain in a small proportion of cases [12, 13, 16]. The characteristics include gradual onset, starting with scrotal swelling followed by pain, [11, 12] as well as complaints such as malaise, fever, and chills are common in epididymal TB patients [12, 13]. Our patient also complained about a non-remitting cough for over 2 months and poor weight gain for 1 year, suggesting TB symptoms. Physical examination may show an enlarged, irregular, nodular and tender epididymis [6, 12]. Most cases are unilateral, although bilateral association may occur in about 30% and is often associated with male infertility due to obstruction of the epididymal tubules [12, 13]. 

Supportive examinations needed to establish epididymal TB include PPD test, CXR, as well as bacteriological evidence in sputum and urine. Urinalysis usually shows microscopic hematuria or sterile pyuria, which increases the suspicion of TB, although it is not a definitive test for GUTB. PPD test and bacteriologic examination did not support the diagnosis of TB, but CXR showed perihilar lymphadenopathy. Bacteriologic tests such as AFB smear, GeneXpert MTB/Rif, and *M. tuberculosis* culture of sputum and urine are essential, although the chances of obtaining positive results are minimal. A study conducted in China on epididymal-orchitis adults patient mentioned that only 15.3% showed positive AFB smear, with 55.8% and 16.3% having positive GeneXpert MTB/Rif from tissue, and positive *M. tuberculosis* culture results respectively [10]. Bacteriological examinations from urine are the gold standard in diagnosing GUTB, but low sensitivity of culture examination has been reported by some literatures. Therefore, negative culture results do not necessarily exclude the possibility of epididymal TB [11]. In this report, urinalysis results were within normal limits, urine culture was sterile, and there was no bacteriological evidence of *M. tuberculosis*.

The differential diagnosis of the enlarged scrotum in children is a primary testicular tumor, bacteria epididymal-orchitis, or metastasis [15]. The patient was evaluated for malignancy with tumor markers and radiology. The results of tumor markers including AFP, beta-HCG, and LDH, as well as radiology namely testicular US and MRI showed no malignancy. Furthermore, another crucial differential diagnosis of epididymal TB is pyogenic epididymitis. The difference between both lies in the onset, disease course and radiologic features. In epididymal TB, symptoms are milder, chronic, and gradual compared to epididymitis pyogenic, hence, patients often report late for treatment. Epididymitis pyogenic is more often acute in onset, although this feature can also be found in epididymal TB at 66.7% [5].

US in epididymal TB can be an inhomogeneous enlargement of hypoechoic epididymitis, while epididymitis pyogenic tends to be homogeneous [12, 17]. In this case, US of the testicles showed a thickening of the scrotal wall with a collection of septae fluid, the size of the left epididymis was slightly enlarged, the texture of the parenchyma was inhomogeneous, and there was no mass. Inhomogeneous features are more common in TB than in epididymitis pyogenic [17]. In addition, in epididymal TB, there is often a thickening of the scrotal wall accompanied by the formation of sinuses [17]. The patient also showed a thickening of the scrotal wall, but no sinuses were formed. Epididymal TB can progress to the testis, which should be suspected when a large epididymal mass or abscess is found [12]. However, there was no signs of abscess or scrotal fistula, which could be one of the clinical complications of epididymal TB.

Imaging modalities commonly used to confirm epididymal TB include US, computed tomography scan (CT-Scan) or MRI of the scrotum. These modalities can show diffuse or focal heterogeneous lesions in the enlarged epididymis, with or without hydrocele, the presence of septae, extra testicular calcification, scrotal abscess, or scrotal sinus tract [18, 19]. Despite US being cheap, easy to perform, associated with minimal risk of radiation, and having remarkable reliability for assessing complications of epididymal TB, the examination has low specificity for the diagnosis of epididymal TB [17]. US is the gold standard for evaluating the scrotum, but not for epididymal TB [20]. Besides, renal US is necessary to evaluate the upper urinary tract for hydronephrosis frequently found in GUTB with prostate association [12]. On MRI, TB lesions may show a T2 hypointense picture [21]. In the patient, MRI examination of the testis presented an enlarged left testis with inhomogeneous hyperintense signal intensity changes on T1WI, inhomogeneous hypointense on T-weighted image (TWI), and T2 STIR, as well as partially restricted diffusion on DWI-ADC. On post-contrast scan, the examination appeared to give inhomogeneous enhancement, suggesting TB over epididymal pyogenic.

Aside from bacteriological evidence, another definitive examination of epididymal TB is fine needle aspiration biopsy (FNAB) cytology or surgical resection of the epididymis [22]. FNAB plays an essential role in excluding the differential diagnosis of epididymal nodules, such as malignancy, or other acute and chronic benign tumors [12, 21, 22]. This modality can provide an accurate and rapid diagnosis as well as prevent aggressive and potentially inappropriate surgical procedures [12, 22]. However, invasive biopsy is often not feasible, leaving only radiological examination establish the diagnosis of epididymal TB. Biopsy is also contraindicated in painless scrotal tumors due to the risk of lymphatic spread of malignant cells during aspiration in suspected malignant tumors [6, 11, 12]. In this patient, neither FNAB nor open biopsy could be performed because testicular FNAB was not feasible at our hospital, and the lesion size shrunk after being given intensive phase therapy of ATT for 2 months.

Epididymal TB can be cured with ATT consisting of isoniazid (H), rifampicin (R), pyrazinamide (Z), and ethambutol (E) given daily for 6–9 months [12, 23, 24]. The 9−12 months treatment is required only in complex cases such as comorbid, recurrent, immunosuppressed status, and HIV patients [12, 13]. Surgical interventions such as epididymal-orchiectomy may be necessary to treat abscesses, obstruction, and failure of conventional therapy [121]. 2RHZE/4RH therapy was given to the patient because isolated epididymal TB had no complications, and there was clinical improvement in the form of a disappearing cough, weight gain of 5 kg, and decreased testicular size with no signs of inflammation.

According to the previous report, the main complication of epididymal TB is infertility [12]. *M. tuberculosis* infection may not directly decrease sperm motility and viability, but infertility potentially results from the blockage of the genital tract at any level [12]. In this context, semen analysis is a laboratory test performed to assess fertility in prostate TB [12]. Significant leukocytopenia can also be found in patients with prostate TB [12]. Sperm analysis was conducted and the results suggested teratozoospermia, Teratozoospermia is defined as morphological abnormalities in more than 85% of sperm, resulting from impaired cell differentiation during spermatogenesis associated with several genetic and environmental factors [25]. Various medical conditions, potentially increase systemic and seminal oxidative stress that may affect sperm count, motility, and morphology, leading to interference with spermatogenesis [25]. Aside from infertility, complications of epididymal TB also include sexual dysfunction and renal failure. A study conducted in China showed that postoperative complications include sexual dysfunction (4.9%), premature ejaculation (6.2%), and infertility (8.6%) [11]. Delayed diagnosis results in disease progression and potential complications such as urethral stricture, contracted bladder, obstructive nephropathy, renal parenchymal damage, and renal failure [26]. In the patient, kidney function test (blood urea nitrogen/ BUN), creatinine, glomerular filtration rate (GFR), or renal US results were in normal limits.

The definitive diagnosis of epididymal TB is based on bacteriological confirmation and histopathology results of the lesions, but implementation in the clinical field has proven to be a challenge. The limitation of this case report is that we did not perform an open biopsy on this patient due to clinical consideration that there was an improvement after the intensive phase of ATT. In addition, urine lipoarabinomannan (LAM) not performed following World Health Organization (WHO) guidelines, which recommended the test for only HIV patients. TB should be suspected in cases of gradual chronic epididymitis that does not respond to antibiotics, specifically when the testicle US results support already established features. Treatment with quadruple ATT for 6–9 months should be the first choice in the management of simple epididymal TB. Complications can include infertility, sexual dysfunction, and renal failure, hence, long-term monitoring may be needed, specifically when among children.

## Data Availability

No datasets were generated or analysed during the current study.

## Abbreviations

AFB: Acid-fast bacilli
AFP: Alpha-fetoprotein
ATT: Anti-tuberculosis therapy
CEA: Carcinoembryonic antigen
CT-Scan: Computed tomography scan
CXR: Chest x-ray
FDC: Fixed dose combination
FNAB: Fine needle aspiration biopsy
GFR: Glomerular filtration rate
GUTB: Genitourinary Tuberculosis
LDH: Lactate dehydrogenase
MRI: Magnetic resonance imaging
MTB: *Mycobacterium tuberculosis*
PPD: Purified protein derivative
qid: quater in die/ four times a day
TB: Tuberculosis
US: Ultrasound
